# Bright Spots and Blind Spots: Three Decades of Cumulative Dis/Advantage Research and What We’re Still Getting Wrong

**DOI:** 10.1093/geroni/igaf122.561

**Published:** 2025-12-31

**Authors:** Jessica Kelley

**Affiliations:** Case Western Reserve University, Cleveland, Ohio, United States

## Abstract

In the canon of theories that are central to understanding the social dynamics of aging, cumulative dis/advantage is one of the most widely cited. Its introduction and early application in the field by scholars Dannefer, Crystal, and others stood in stark contrast to other common frameworks such as normative aging, stress process, person-environment fit. CDA centrally focuses on social inequality that operates at micro, meso, and macro levels of society and shapes life chances of individuals from birth to death. The structural and systemic features in the allocation of resources creates a predictable set of processes, whereby the (lack of) resources becomes a central predictor in access to future resources. Empirical evidence has affirmed increasing inequality over the life course. Despite its wide-ranging explanatory potential, CDA has too often been limited in its application by researchers. Its intuitive appeal in explaining how accumulating adversity can lead to a cascade of negative outcomes as a person ages (and vice versa for accumulating advantages) can often be the only feature of the theory that is highlighted or tested. Studies can often be testing for “chains of risk” that occur as one ages rather than the systemic inequality that perpetuates the unequal opportunity structure. Likewise, focusing on social mediators that may lessen the impact of early-life adversity looks to individual-level solutions to a structural problem. I will discuss the state of the field and offer potential new avenues to expand the application of cumulative dis/advantage theory in gerontology.

